# Association Between Generalized Anxiety Disorder Scores and Online Activity Among US Adults During the COVID-19 Pandemic: Cross-Sectional Analysis

**DOI:** 10.2196/21490

**Published:** 2020-09-10

**Authors:** Parvati Singh, William G Cumberland, Dominic Ugarte, Tim-Allen Bruckner, Sean D Young

**Affiliations:** 1 University of California Institute for Prediction Technology University of California, Irvine Irvine, CA United States; 2 Fielding School of Public Health University of California, Los Angeles Los Angeles, CA United States; 3 School of Medicine, University of California, Irvine Irvine, CA United States; 4 Program in Public Health University of California, Irvine Irvine, CA United States

**Keywords:** online activity, COVID-19, anxiety, generalized anxiety disorder, GAD, scores, stress, anxiety, internet, survey, cross-sectional

## Abstract

**Background:**

Evidence from past pandemics suggests that fear, uncertainty, and loss of control during large-scale public health crises may lead to increased pandemic-related information seeking, particularly among persons predisposed to high anxiety. In such groups, a greater consumption of information pertaining to the COVID-19 pandemic may increase anxiety.

**Objective:**

In this study, we examine the association between online activity and Generalized Anxiety Disorder 7 (GAD-7) scores in the United States.

**Methods:**

We recruited participants for an online survey through advertisements on various platforms such as Google, Facebook, and Reddit. A total of 406 adult US participants with moderate to severe (≥10) GAD-7 scores met the inclusion criteria and completed the survey. Anxiety levels measured using the GAD-7 scale formed our primary outcome. Our key independent variables were average daily time spent online and average daily time spent online searching about COVID-19 within the past 14 days. We used as controls potential confounders of the relation between our key independent variables and GAD-7 scores, namely, sleep quality, the COVID-19 Fear Inventory scale, binge drinking, substance use, prescription drug abuse, and sociodemographic attributes.

**Results:**

Linear multivariate regression analyses showed that GAD-7 scores were higher among those who spent >4 hours online (per day) searching for information about COVID-19 (coefficient 1.29, *P*=.002), controlling for all other covariates. The total time spent online was not statistically associated with GAD-7 scores.

**Conclusions:**

Results from this study indicate that limiting pandemic-related online information seeking may aid anxiety management in our study population.

## Introduction

The COVID-19 pandemic has impacted multiple aspects of everyday life across the world. Social distancing measures mandated by state governments in the United States, including stay-at-home orders and closure of nonessential businesses, have preceded record-breaking unemployment rates and an unprecedented US $3 trillion relief and economic stimulus package from Congress [1-3]. These sweeping changes have altered daily routines of the US population and may correspond with marked changes in mental and behavioral health, exacerbated by social isolation, fear, uncertainty, and financial strain during this pandemic [4].

The convergence of social isolation, heightened ambient stress, and potential loss of resources including access to health care has elicited calls for research on correlates of mental health outcomes amid the current pandemic [5]. Understandably, COVID-19 has spurred a substantial amount of research on mental health in the present times. According to a recent meta-analytic review, populations across multiple geographies worldwide have experienced an approximately 30% increase in anxiety, depression, and stress [6]. These reports cohere with mental distress observed in the United States [7]. About one-third of the adult population in the United States has reported increased anxiety, trauma, depression, and substance use in the months following the COVID-19 pandemic [7]. Approximately 1 in 10 US adults have also reported having seriously contemplated suicide [7].

The initial stages of pandemics invariably correspond with widespread anxiety [8]. Scholars examining past pandemics report overconsumption of information (and misinformation) from 24-hour news cycles and social media as potential coping mechanisms among populations faced with fear and uncertainty [8]. Individual-level psychological responses to pandemics may vary by personal intolerance to uncertainty thresholds [8,9]. For instance, during the 2014 swine flu epidemic, people with high intolerance to uncertainty were also more likely to report fear and anxiety about getting infected with the virus, relative to those with lower intolerance [9]. Anxiety correlates positively with intolerance to uncertainty that in turn, may lead to reassurance-seeking behaviors such as frequently checking the internet for medical information or pandemic-related news updates [8]. This maladaptive coping behavior may turn into a self-perpetuating negative feedback cycle. Among persons vulnerable to emotional distress, repeat consumption of information online may thus lead to overestimation of the pandemic threat and exacerbation of anxiety [8].

Research on the mental health impacts of the COVID-19 pandemic in China, which was the first country affected by this pandemic, finds a positive correlation between anxiety and exposure to news and information about COVID-19 online [10]. In one study, a cross-sectional sample of about 4800 online survey participants reported 72% higher odds of anxiety symptoms (but not depression) with frequent exposure to news and information about COVID-19, relative to those with infrequent or low exposure [10]. Another web-based cross-sectional survey of over 7000 people in China also reported an increase in Generalized Anxiety Disorder 7 (GAD-7) scores with more than 3 hours (per day) spent discussing or searching about COVID-19 [11]. Huang and Zhao [11] observed poorer sleep quality- a strong correlate of anxiety and depression- among health care workers, in alignment with contemporaneous reports of adverse mental health outcomes in this occupational group [12]. Research examining trends in mental health–related search terms on Google across multiple countries also reported an increase in search volume for “insomnia” immediately following the current pandemic in those regions [13].

Online studies are increasingly becoming a popular means of conducting health research during the COVID-19 pandemic [14-16]. Online surveys have wide reach, as a large majority of the population has rapidly gained access to the internet over the past decade with over 80% of households in the United States reporting having an internet-connected computer or smartphone [17]. Quarantine and social distancing measures currently in effect owing to the COVID-19 pandemic make online surveys particularly lucrative for data collection and research [15,16].

This study examines the relation between GAD-7 scores and time spent online searching for information about COVID-19 in a cross-section of 406 online survey participants with moderate to severe anxiety in the United States. To our knowledge, this is the first study to examine the relation between online activity pertaining to COVID-19 and GAD-7 scores in a US sample, and may help augment our understanding of the mental health consequences of the current pandemic.

## Methods

### Participant Recruitment

We recruited study participants through online advertisements on Facebook, Google ads, and online forums such as Reddit. Potential participants who clicked on the online ads were routed to a Qualtrics survey where they were screened for eligibility using the GAD-7 screening tool, which we modified in relation to COVID-19 (for the detailed modified GAD-7 instrument, please see the first table in Multimedia Appendix 1). To be eligible for participation, participants needed to meet the following inclusion criteria: adults 18 years or older who were competent enough to give informed consent, English speakers, moderate to severe GAD-7 scores (≥10) in relation to COVID-19, not currently taking anxiety medication, uses social media or online communities greater than twice per week, willing and capable of understanding and assenting to an online informed consent form, has or is willing to accept a group invitation from our Facebook social media page, and has completed the survey.

Those who did not meet all items on the inclusion list were ineligible for the study. After eligible participants provided their consent online and liked or messaged our study’s Facebook page, they were invited to complete our study survey, for which they received US $15 Amazon gift cards post survey completion. Given that persons with pre-existing anxiety appear differentially exposed and vulnerable to online information seeking during pandemics, we selected persons who have moderate to severe anxiety (ie, ≥10 GAD-7 score) [18] to have a sufficiently large sample size of persons with high anxiety for our study.

The survey was conducted from March 15, 2020, to April 8, 2020. University of California, Irvine’s Institutional Review Board (IRB) deemed this study as IRB exempt. Our analytic sample comprised of 406 respondents who met the inclusion criteria and responded to all items on the survey.

### Variables

For the dependent variable, we measured anxiety levels using the GAD-7 scale [18]. Scores on the GAD-7 scale are determined through participant responses to a 7-item anxiety questionnaire. The resulting (additive) GAD-7 score was defined as the outcome of interest.

The key independent variables included time spent online (6 categories: none, 1 minute to 2 hours, 2-4 hours, 4-6 hours, 6-8 hours, >8 hours) and time spent online searching about COVID-19 (6 categories: none, 1 minute to 2 hours, 2-4 hours, 4-6 hours, 6-8 hours, >8 hours). We converted time spent online searching about COVID-19 from categorical to binary (≤4 hours per day or >4 hours per day) for clarity of interpretation (very few participants reported spending <2 hours searching about COVID-19 per day).

As described previously, pandemics correspond with a decline in quality of sleep, increased pandemic fear, and increased substance use [7,8]. These factors may drive greater online information seeking or serve as mediators of the relation between time spent online and increased anxiety. To that end, we included as our control variables binge drinking (binary: <5 or ≥5 alcoholic drinks in any day for males, ≥4 alcoholic drinks in any day for females, in the past 14 days), drug use (eg, marijuana, cocaine, heroin), and prescription drug abuse (eg, opioids, Adderall) in the past 14 days (binary: yes or no). We also derived a sleep scale using responses to items adapted from the Medical Outcomes Study (MOS) scale through Cronbach alpha and used this continuous variable as an exposure (α=.78; second table in Multimedia Appendix 1) [19]. In addition, we used a validated pandemic fear inventory scale and defined a COVID-19 Fear Inventory scale using Cronbach alpha estimates from 10 item responses (α=.76; third table in Multimedia Appendix 1) [20]. Inclusion of these covariates allows us to estimate the relation between our dependent and key independent variables “net of” plausible and measurable confounding factors. Other control variables included education (binary: <high school or ≥high school), sex (male, female), race (non-Hispanic White, non-Hispanic Black, Hispanic, Asian, others), and age (in years).

### Statistical Analysis

We used ordinary least squares multivariate regression analysis to examine the relation between GAD-7 scores and the independent, control variables. We specified robust standard errors to account for heteroskedasticity. Regression coefficients with *P*<.05 were deemed statistically significant. All analyses were performed in Stata SE version 14.2 (StataCorp) [21]. There were no missing data in our analytic data set, as all 406 participants completed the survey in entirety.

## Results

Table 1 shows the descriptive statistics of the variables included in this study. The average GAD-7 score among the study participants was 17.2, and mean scores on the MOS sleep scale and COVID-19 Fear Inventory scale were 2.2 and 5, respectively. About 83% (336/406) of the participants reported greater than 4 hours spent searching for information about COVID-19 online per day in the past 14 days. The mean age of study participants was approximately 39 years, with 82% (332/406) females and 18% (74/406) males. Approximately 75% (307/406) were non-Hispanic White, 7% (28/406) non-Hispanic Black, and 10% (41/406) Hispanic. Most participants reported having a high school or General Educational Development (GED), or higher educational attainment (394/406, 97%).

**Table 1:** Descriptive statistics of variables included in study (N=406).

Variables		Participants
GAD-7^a^ score, mean (SD)		17.2 (3.2)
MOS^b^ Sleep Scale score, mean (SD)		2.2 (0.9)
COVID-19 Fear Inventory score, mean (SD)		4.2 (0.5)
Age (years), mean (SD)		38.9 (12.7)
**Time spent online per day in the past 14 days, n (%)**		
	None	0 (0.0)
	1 minute to 2 hours	6 (1.5)
	2-4 hours	62 (15.27)
	4-6 hours	103 (25.37)
	6-8 hours	107 (26.35)
	>8 hours	128 (31.53)
Greater than 4 hours daily spent online searching for/reading information/watching videos about COVID-19 (eg, causes of it, symptoms, effects of COVID-19 on society/the stock market/employment issues, news reports about it) in the past 14 days, n (%)		336 (82.8)
Binge drinking on any day within the past 14 days, n (%)		98 (24.1)
Used any drugs including marijuana, cocaine or crack, heroin, methamphetamine (crystal meth), hallucinogens, ecstasy/MDMA in the past 14 days, n (%)		69 (17)
Used any prescription medications just for the feeling, more than prescribed, or that were not prescribed (eg, OxyContin, Vicodin, Percocet, Methadone, Xanax, Ativan, Klonopin, Adderall, or Ritalin), n (%)		20 (4.9)
**Race/ethnicity, n (%)**		
	Non-Hispanic White	307 (75.6)
	Non-Hispanic Black	28 (6.9)
	Hispanic	41 (10.1)
	Asian	14 (3.45)
	Other	16 (3.94)
Greater than or equal to high school/GED^c^ education, n (%)		394 (97)
**Sex, n (%)**		
	Female	332 (81.8)
	Male	74 (18.2)

^a^GAD-7: Generalized Anxiety Disorder 7.

^b^MOS: Medical Outcomes Study.

^c^GED: General Educational Development.

Table 2 shows the results from the linear regression analysis predicting GAD-7 scores as a function of covariates included in the study. The time spent in daily online activity was not statistically associated with GAD-7 scores. However, more than 4 hours of daily online activity searching for information about COVID-19 was associated with higher GAD-7 scores (coefficient 1.29, *P*=.002). This relation remained robust when using the 6-category (none, 1 minute to 2 hours, 2-4 hours, 4-6 hours, 6-8 hours, >8 hours) version of daily online activity searching for information about COVID-19 as well (results available upon request). The sleep scale scores varied inversely (coefficient –1.02, *P*<.001), and the COVID-19 Fear Inventory scale scores varied positively and significantly (coefficient 1.46, *P*<.001) with GAD-7 scores. Misuse or abuse of prescription drugs in the past 14 days was associated with higher GAD-7 scores (coefficient 1.12, *P*=.007). Participants with high school or GED, or higher education showed lower GAD-7 scores relative to those without high school education (coefficient –2.77, *P*<.001). Relative to non-Hispanic Whites, GAD-7 scores did not vary across non-Hispanic Blacks, Hispanics, Asians, and other races or ethnicities. We also observed no relation between GAD-7 scores and age, sex, alcohol use, and use of nonprescription drugs such as marijuana, cocaine, or heroin.

**Table 2:** Multivariate linear regression results predicting Generalized Anxiety Disorder 7 score as a function of key covariates.

Covariates		Coefficient (95% CI)	*P* value
**Time spent online per day in the past 14 days (reference: 1 minute to 2 hours)**			
	2-4 hours	–0.59 (–2.54 to 1.36)	.55
	4-6 hours	–0.05 (–1.95 to 1.85)	.96
	6-8 hours	–0.55 (–2.45 to 1.35)	.57
	>8 hours	–0.03 (–1.92 to 1.87)	.98
Greater than 4 hours online activity searching for/reading information/watching videos about COVID-19 per day		1.29 (0.47 to 2.11)	.002
Consumed more than 5 alcoholic drinks a day (in the past 14 days)		0.31 (–0.32 to 0.93)	.34
Used any drugs including marijuana, cocaine or crack, heroin, methamphetamine (crystal meth), hallucinogens, ecstasy/MDMA in the past 14 days		–0.18 (–0.94 to 0.57)	.64
Used prescription medications just for the feeling, more than prescribed, or that were not prescribed (eg, opiates, medications for anxiety, sleep, ADHD^a^) in the past 14 days		1.12 (0.30 to 1.93)	.007
MOS^b^ Sleep Scale score		–1.02 (–1.35 to –0.69)	<.001
COVID-19 Fear Inventory Scale score		1.47 (0.76 to 2.17)	<.001
Age		0.00 (–0.03 to 0.02)	.91
Female (reference: male)		0.66 (–0.12 to 1.45)	.10
Greater than or equal to high school/GED^c^ education		–2.77 (–4.10 to –1.44)	<.001
**Race/ethnicity (reference: non-Hispanic White)**			
	Non-Hispanic Black	1.24 (–0.89 to 1.12)	.82
	Hispanic	1.43 (–0.72 to 1.22)	.61
	Asian	1.49 (–0.33 to 2.60)	.13
	Other	–1.25 (–2.55 to 0.05)	.06
Sample size (N)		406	N/A^d^
Adjusted R^2^		0.221	N/A

^a^ADHD: attention-deficit/hyperactivity disorder.

^b^MOS: Medical Outcomes Study.

^c^GED: General Educational Development.

^d^N/A: not applicable.

## Discussion

### Principal Results

We found that spending an average of 4 or more hours per day searching online about COVID-19 was associated with greater anxiety (measured using GAD-7 scores) in our cross-sectional sample of 406 US adults with moderate to severe anxiety. Relative to those who spent less than 4 hours on pandemic-related online searches, those who exceeded 4 hours of COVID-19–related online activity had GAD-7 scores that were 1.3 units higher. Our analyses controlled for individual sociodemographic attributes, time spent online overall, substance use, binge drinking, sleep quality, and the COVID-19 Fear Inventory. Our findings align with those from prior research and highlight the relation between online information seeking and worsened anxiety symptoms in the context of the COVID-19 pandemic [8,10,11].

### Limitations

Limitations include the cross-sectional nature of our study design that restricts causal inference and generalizability. We also note that, although GAD-7 scores may be used to assist with a psychiatric diagnosis of generalized anxiety disorder, this study uses them to approximate anxiety levels (over the past 2 weeks preceding the survey only) and not as a clinical diagnosis. Clinical diagnosis of generalized anxiety disorder, per the Diagnostic and Statistical Manual of Mental Disorders, 5th Edition criteria, typically requires continued observation over a period of 6 months [18]. For the purposes of this study, GAD-7 scores are well suited to capture proximate responses to a sudden, exogenous, and acute stressor (ie, the COVID-19 pandemic).

### Implications for Future Research

We encourage future research to examine whether (as indicated by our findings) relatively simple measures such as limiting exposure to COVID-19–related information may help in managing anxiety during pandemics. Our study may aid future research in examining the effect of misinformation during pandemics because persons exposed to a higher volume of COVID-19–related information may also, statistically speaking, be exposed to greater misinformation (owing to the sheer volume of exposure). People with high anxiety and high intolerance to uncertainty, when exposed to quack cures and misinformation, may be more likely to act on potentially fatal “preventive” measures [8,22]. Curation of accurate information available online (eg, fact-checking by scientific organizations) may serve as an effective tool in preventing adverse outcomes [23].

It is plausible that persons with high anxiety *select into* greater pandemic-related information seeking online. In such cases, the propensity of persons with high anxiety to spend substantial amounts of time online may be used in targeted internet-based mental health service delivery. High frequency internet and social media users with pre-existing mental health conditions may serve as an accessible group for telepsychiatry, telemedicine, and social media–based interventions [5]. Such interventions and services offer the dual benefit of reaching target (ie, high anxiety) populations while adhering to pandemic prevention guidelines (eg, lower interpersonal contact, social distancing) [5].

### Conclusion

In this cross-sectional study, we observed a positive relation between time spent online searching for information about COVID-19 and GAD-7 scores. Our analyses may assist in understanding correlates of mental health outcomes during the ongoing COVID-19 pandemic.

## Appendix

Multimedia Appendix 1 Supplementary material.

## Abbreviations

GAD-7: Generalized Anxiety Disorder 7
IRB: Institutional Review Board
MOS: Medical Outcomes Study
