# Supplementary material for: Association Between Generalized Anxiety Disorder Scores and Online Activity Among US Adults During the COVID-19 Pandemic: Cross-Sectional Analysis
Source: J Med Internet Res. 2020 Sep 10;22(9):e21490. doi: 10.2196/21490 (PMC7485999; doi:10.2196/21490)
Supplement: Multimedia Appendix 1 [file jmir_v22i9e21490_app1.docx]

**Supplementary File**

**Supplementary Table 1:** Generalized Anxiety Disorder-7 survey items (modified for COVID-19)

“Please answer the following questions specifically in relation to your thoughts/concerns about the Coronavirus. This can include fears of contracting the virus, spreading it, missing work/income because of it, having to be quarantined/miss out on opportunities, losing money in the stock/housing/financial markets, and any other issues that might be related to the virus, news report, and communications about it.

During the last 2 weeks, how often have you been bothered by the following problems related to the Coronavirus? For each question, please select an answer from 0-3 (0 = Not at all, 1 = Several days, 2 = Over half the days, 3 = Nearly every day).”

| Over the last 2 weeks, how often have you been bothered by the following problems?   1. Feeling nervous, anxious, or on edge 2. Not being able to stop or control worrying 3. Worrying too much about different things 4. Trouble relaxing 5. Being so restless that it's hard to sit still 6. Becoming easily annoyed or irritable 7. Feeling afraid as if something awful might happen   *Add the score for each column*  Total Score *(add your column scores)* = | Not at | Several | Over half | Nearly |
| --- | --- | --- | --- | --- |
|  | all sure | days | the days | every day |
|  | 0 | 1 | 2 | 3 |
|  | 0 | 1 | 2 | 3 |
|  | 0 | 1 | 2 | 3 |
|  | 0 | 1 | 2 | 3 |
|  | 0 | 1 | 2 | 3 |
|  | 0 | 1 | 2 | 3 |
|  | 0 | 1 | 2 | 3 |
|  | + | + | + |  |
|  |  | | | |

**Supplementary Table 2:** Survey questions and item properties of Medical Outcomes Study (MOS) Sleep Scale

| **Survey question/Item** | **Obs** | **Sign** | **item-test correlation** | **item-rest correlation** | **average inter-item covariance** | **alpha** |
| --- | --- | --- | --- | --- | --- | --- |
| On average, how long did it take for you to fall asleep during the past 14 days? Pick one answer. | 406 | + | 0.3511 | 0.1796 | 0.50194 | 0.7723 |
| On average, how many hours did you sleep each night during the past 14 days? | 406 | + | 0.3002 | 0.2028 | 0.529831 | 0.7677 |
| On average, how many hours did you feel that your sleep was not quiet (moving restlessly, feeling tense, speaking, etc., while sleeping)? | 377 | + | 0.4745 | 0.3424 | 0.477133 | 0.7542 |
| On average, how many hours did you get enough sleep to feel rested upon waking in the morning? | 300 | - | 0.3921 | 0.2313 | 0.510104 | 0.7771 |
| On average, how many hours did you awaken short of breath or with a headache? | 317 | + | 0.4919 | 0.3204 | 0.463967 | 0.7597 |
| On average, how many hours did you feel drowsy or sleepy during the day and have trouble falling asleep? | 357 | + | 0.4776 | 0.3446 | 0.460871 | 0.7469 |
| On average, how many hours did you waken during your sleep time and have trouble falling asleep again? | 353 | + | 0.4566 | 0.3037 | 0.465678 | 0.7545 |
| On average, how many hours did you have trouble staying awake during the day? | 286 | + | 0.6068 | 0.4726 | 0.429835 | 0.7399 |
| On average, how many hours did you snore during your sleep? | 325 | + | 0.4596 | 0.2451 | 0.496597 | 0.7892 |
| On average, how many hours did you take naps (5 minutes or longer) during the day? | 322 | + | 0.4438 | 0.2462 | 0.485662 | 0.7776 |
| On average, how many hours did you get the amount of sleep you needed? | 289 | - | 0.5926 | 0.4629 | 0.432574 | 0.7392 |
|  |  |  |  |  |  |  |
| MOS Sleep Scale |  |  |  |  | 0.477242 | 0.7788 |

**Supplementary Table 3:** Survey questions and item properties of COVID-19 Fear Inventory (CFI) Scale

| **Survey question/Item** | **Obs** | **Sign** | **item-test correlation** | **item-rest correlation** | **average inter-item covariance** | **alpha** |
| --- | --- | --- | --- | --- | --- | --- |
|  |  |  |  |  |  |  |
| To what extent are you concerned about coronavirus? | 400 | + | 0.6788 | 0.5919 | 0.153978 | 0.7308 |
| To what extent do you believe that coronavirus could become a "pandemic" in the U.S.? | 405 | + | 0.4649 | 0.4221 | 0.181283 | 0.7586 |
| How likely is it that you could become infected with coronavirus? | 406 | + | 0.6449 | 0.4962 | 0.141592 | 0.7328 |
| How likely is it that someone you know could become infected with coronavirus? | 406 | + | 0.6496 | 0.5124 | 0.142792 | 0.7305 |
| How quickly do you believe contamination from coronavirus is spreading in the U.S.? | 406 | + | 0.6566 | 0.5532 | 0.148843 | 0.7276 |
| If you did become infected with coronavirus, to what extent are you concerned that you will be severely ill? | 406 | + | 0.5817 | 0.3903 | 0.146914 | 0.7551 |
| To what extent has the threat of coronavirus influenced your decisions to be around people? | 403 | + | 0.6546 | 0.5466 | 0.150082 | 0.7305 |
| To what extent has the threat of coronavirus influenced your travel plans? | 397 | + | 0.5734 | 0.4019 | 0.152352 | 0.7507 |
| To what extent has the threat of coronavirus influenced your use of safety behaviors (e.g., hand sanitizer)? | 403 | + | 0.6067 | 0.5015 | 0.156145 | 0.7369 |
| How knowledgeable to you feel about coronavirus? | 399 | + | 0.3646 | 0.1945 | 0.174388 | 0.7748 |
|  |  |  |  |  |  |  |
| CFI scale |  |  |  |  | 0.154844 | 0.763 |
